# Assessing the readability, quality and reliability of responses produced by ChatGPT, Gemini, and Perplexity regarding most frequently asked keywords about low back pain

**DOI:** 10.7717/peerj.18847

**Published:** 2025-01-22

**Authors:** Erkan Ozduran, Volkan Hancı, Yüksel Erkin, İlhan Celil Özbek, Vugar Abdulkerimov

**Affiliations:** 1 Physical Medicine and Rehabilitation, Pain Medicine, Sivas Numune Hospital, Sivas, Turkey; 2 Anesthesiology and Reanimation, Critical Care Medicine, Dokuz Eylül University, Izmir, Turkey; 3 Anesthesiology and Reanimation, Pain Medicine, Dokuz Eylül University, Izmir, Turkey; 4 Physical Medicine and Rehabilitation, Health Science University, Derince Education and Research Hospital, Kocaeli, Turkey; 5 Anesthesiology and Reanimation, Central Clinical Hospital, Baku, Azerbaijan

**Keywords:** Artificial intelligence, ChatGPT, Gemini, Low back pain, Online medical information, Perplexity

## Abstract

**Background:**

Patients who are informed about the causes, pathophysiology, treatment and prevention of a disease are better able to participate in treatment procedures in the event of illness. Artificial intelligence (AI), which has gained popularity in recent years, is defined as the study of algorithms that provide machines with the ability to reason and perform cognitive functions, including object and word recognition, problem solving and decision making. This study aimed to examine the readability, reliability and quality of responses to frequently asked keywords about low back pain (LBP) given by three different AI-based chatbots (ChatGPT, Perplexity and Gemini), which are popular applications in online information presentation today.

**Methods:**

All three AI chatbots were asked the 25 most frequently used keywords related to LBP determined with the help of Google Trend. In order to prevent possible bias that could be created by the sequential processing of keywords in the answers given by the chatbots, the study was designed by providing input from different users (EO, VH) for each keyword. The readability of the responses given was determined with the Simple Measure of Gobbledygook (SMOG), Flesch Reading Ease Score (FRES) and Gunning Fog (GFG) readability scores. Quality was assessed using the Global Quality Score (GQS) and the Ensuring Quality Information for Patients (EQIP) score. Reliability was assessed by determining with DISCERN and Journal of American Medical Association (JAMA) scales.

**Results:**

The first three keywords detected as a result of Google Trend search were “Lower Back Pain”, “ICD 10 Low Back Pain”, and “Low Back Pain Symptoms”. It was determined that the readability of the responses given by all AI chatbots was higher than the recommended 6th grade readability level (*p* < 0.001). In the EQIP, JAMA, modified DISCERN and GQS score evaluation, Perplexity was found to have significantly higher scores than other chatbots (*p* < 0.001).

**Conclusion:**

It has been determined that the answers given by AI chatbots to keywords about LBP are difficult to read and have low reliability and quality assessment. It is clear that when new chatbots are introduced, they can provide better guidance to patients with increased clarity and text quality. This study can provide inspiration for future studies on improving the algorithms and responses of AI chatbots.

## Introduction

Low back pain (LBP) is a very common symptom and affects people of almost every age group. It is stated that the point prevalence of LBP that limits activity is 7.3% and 540 million people suffer from this complaint at some point in their lives. Not only that, it is emphasized that LBP is the number one cause of disability globally (Hartvigsen et al., 2018). In a study conducted by the Journal of the American Medical Association, it was determined that the expenditure on spine-related problems is the most costly expenditure after diabetes and heart disease. While medications, invasive procedures, imaging, and surgeries constitute direct related costs, disability, loss of productivity, and loss of wages are stated as indirect costs (Hemmer, 2021). The causes of LBP can often be mechanical, as well as chronic inflammatory diseases such as ankylosing spondylitis, which affects a rate of 0.1–1.4% of the population (Bagcier, Yurdakul & Ozduran, 2021). According to the Centers for Disease Control and Prevention in the USA, in 2016, there were 3.6 million visits to emergency departments and 5.7 million visits to urgent and ambulatory care clinics due to back-related complaints (DePalma, 2020). Infection, fracture or trauma, malignancy, *etc*., are conditions that suggest urgent pathology. These conditions are called red flags, and failure to diagnose this condition by clinicians can lead to delayed treatment and increased patient morbidity and mortality (Verhagen et al., 2016).

Artificial intelligence (AI) can be defined as the study of algorithms that provide machines with the ability to reason and perform cognitive functions, including object and word recognition, problem solving, and decision making (Grippaudo et al., 2024). Artificial intelligence has gained popularity in recent years. Studies in the literature emphasize that the use of artificial intelligence robots that enable people to interact with technology in a more social and conversational manner is increasing (Grippaudo et al., 2024; Gül et al., 2024). An example of conversational artificial intelligence is ChatGPT, developed by OpenAI (San Francisco, CA, USA). It is widely used in many fields, especially in medical fields, and its reliability and effectiveness have been evaluated in many studies (Gül et al., 2024; Şahin et al., 2024). Perplexity AI is an artificial intelligence model that provides answers to queries and directions and includes links to quotations and related topics, while Google Gemini is an artificial intelligence model capable of analyzing complex data sets such as images and graphs (Ömür Arça et al., 2024).

Patients diagnosed with chronic nonspecific LBP do not have sufficient information about the amount and type of physical activity they can perform for their treatment. Therefore, patient education and back school programs for patients with LBP can help with spine protection, rehabilitation and the acquisition of specific information about the disease (Ács et al., 2020; Nolet et al., 2018). As it is known, individuals who have better disease-specific knowledge, accurate information about the cause of the disease, prevention and treatment options have higher rates of protection from the disease and their participation in rehabilitation programs has also been determined to be higher (Járomi et al., 2021). In addition, the acquisition of health information *via* the internet is increasing day by day. In particular, people with LBP constantly express their desire to receive reliable information about their clinical condition (Hodges, Setchell & Nielsen, 2020). Patients can use popular internet search engines as well as artificial intelligence chatbots to obtain information in this area (Yilmaz Muluk & Olcucu, 2024). Artificial intelligence can also be used to monitor and give recommendations to patients experiencing chronic back pain. It can be used as an application that can be installed on mobile devices to monitor patients’ symptoms and activities (Do et al., 2023). Hartmann et al. (2023) found a significant decrease in pain and pain-related impairments in daily living in patients diagnosed with LBP who used the AI-supported exercise application for 8 weeks, compared to the control rehabilitation group that did not use this application.

It is known that technology and the artificial intelligence applications it brings have the potential to increase the quality and safety of healthcare services. However, there are some concerns about the lack of reliability regarding this technology, its inadequate quality and its readability levels that the public can understand (Grippaudo et al., 2024; Gül et al., 2024; Şahin et al., 2024; Ömür Arça et al., 2024). According to the standards determined by the United States Department of Health and Human Services, the American Medical Association and the National Institutes of Health, patient education materials should have a readability grade of six or below (Gül et al., 2024; Ömür Arça et al., 2024; Erkin, Hanci & Ozduran, 2023b; Özduran & Hanci, 2022; Ozduran & Büyükçoban, 2022).

There are increasing number of studies in the literature evaluating the reliability, readability and quality of AI chatbots on low back pathologies. Coraci et al. (2023) studied the development of medical questionnaires for low back pain in ChatGPT. As a result, although they found a significant correlation between other low back pain surveys and the ChatGPT survey, they stated that the power of this artificial intelligence chatbot was limited. Shrestha et al. (2024) studied the performance of ChatGPT in producing a clinical guideline in the diagnosis and treatment of LBP. They found that although ChatGPT provides an adequate clinical guideline recommendation, it tends to incorrectly recommend evidence. Yilmaz Muluk & Olcucu (2024) examined the effectiveness of ChatGPT-3.5 and GoogleBard in detecting Red Flags of LBP. They found that these AI chatbots showed strong performance but contained irrelevant content and showed low sensitivity. Gianola et al. (2024) evaluated the performance of ChatGPT in making informed decisions for lumbosacral radicular pain compared to clinical practice guidelines. They found that ChatGPT performed poorly in terms of internal consistency and accuracy of the generated indications compared to clinical practice guideline recommendations for lumbosacral radicular pain. Nian et al. (2024) searched patient education materials on Lumbar Spinal Fusion and Laminectomy on ChatGPT and Google. They found that ChatGPT responses were longer (340.0 *vs*. 159.3 words) and had lower readability (Flesch-Kincaid grade level: 11.6 *vs*. 8.8, Flesch Reading Ease score: 34.0 *vs*. 58.2) compared to Google. The authors noted that although ChatGPT was able to produce relatively accurate responses to certain questions, its role can be seen as a complement to consultation with a physician and should be used with caution until its functionality is validated (Nian et al., 2024).

Artificial intelligence chatbots have been studied not only on low back pain-related issues but also on different medical subjects, and impressive results have been obtained. Gül et al. (2024) found that the readability levels of Bard, ChatGPT and Perplexity responses to 100 questions related to subdural hematoma were higher than the recommended 6th grade level. They reported that although AI chatbots offer the opportunity to improve health outcomes and patient satisfaction, they are not sufficient in terms of readability. Şahin et al. (2024) evaluated the responses of five different artificial intelligence chatbots named Bard, ChatGPT, Ernie, Bing and Copilot to questions about erectile dysfunction in their study. They found that the AI chatbot that requires a high level of training to be understood is ChatGPT and the chatbot with the easiest readability is BARD. They reported that new AI chatbots to be developed in the future can provide more advanced counseling to patients if their understandability is easier (Şahin et al., 2024).

The increase in online sources of information raises concerns about which sources of information patients can trust and take into account. As mentioned above in the literature, many popular AI chatbots have been discussed in different studies and the information they contain has been analyzed in depth. However, there were no comparative studies of the three most popular AI chatbots on LBP in the literature. In line with this information, this study aimed to examine the quality, reliability, and readability of the responses given by three different AI chatbots (ChatGPT, Gemini, and Perplexity) to frequently asked keywords about LBP.

## Materials and Methods

### Ethics committee permission

This cross-sectional study was prepared after receiving ethics committee approval (Cumhuriyet University Ethics Committee, Ethics Committee No: 2024/05-27, Date: 16.05.2024).

### Research procedure

The research was initiated by deleting all data sets belonging to personal internet browsers. After logging out of Google accounts, the research was continued by activating Google Incognito mode. The most frequently searched keywords related to low back pain were tried to be reached on May 29, 2024 in the Google Trends (https://trends.google.com/) search engine (Hershenhouse et al., 2024). The search criteria were created by selecting health subheadings from all over the world from 2004 to the present. In the results section, the “most relevant” keywords were marked. As a result of the Google trend search, the 25 most frequently searched keywords with different categories were recorded. Geographical areas of interest were classified and recorded on the basis of subregions.

The keywords obtained were entered separately in English to ChatGPT, Gemini and Perplexity AI chatbots, which are freely accessible to everyone (Gül et al., 2024; Currie, Robbie & Tually, 2023). In order to prevent possible bias that could be created by the sequential processing of keywords in the answers given by the programs, the study was designed by providing input from different users (EO, VH) for each keyword. A different user was not assigned for each keyword and fake accounts were not used. The answers were recorded in the database so that they could be examined in terms of readability, reliability and quality. The keywords and responses from each AI chatbots are available from the web archive located at https://archive.org/details/assessing-the-readability-quality-and-reliability-of-responses-produced-by-chat-. Instead of ChatGPT Plus, the study was carried out using the GPT-4o version in the ChatGPT Free AI chatbot, which is free to everyone. In our study, AI chatbots that are freely accessible and accessible to people with low socioeconomic status were used (Gül et al., 2024; Ömür Arça et al., 2024).

### Reliability analysis

The reliability level of the answers was determined in the analysis based on “The Journal of the American Medical Association (JAMA) Benchmark”. In order for a study to meet the JAMA criteria, it must meet four basic criteria such as authorship, currency, disclosure, and attribution. In the evaluation made according to the JAMA criteria, zero or one point is given for each criterion and these points are added up to form a general evaluation of the study between 0 and 4 points. Higher scores indicate that the study is more reliable, while lower scores indicate that it is less reliable (Kara et al., 2024; Ozduran & Hanci, 2023).

Another reliability scale used in our study is the Modified DISCERN scale. In this scale consisting of five criteria, if the required criterion is found, it is represented by 1 point, if not, it is represented by 0 points. Studies evaluated on a 5-point scale are considered more reliable as they receive higher scores (Erkin, Hanci & Ozduran, 2023a).

The questions in the scale can be listed as follows: “Is the literature review based on up-to-date and accurate sources?”, “Are additional information sources listed for patient reference?”, “Does the study address discussions in its field?” “Is the text clear and understandable?”, “Is the information provided balanced and unbiased?” (Erkin, Hanci & Ozduran, 2023b). The validity and reliability of the JAMA and DISCERN scales have been evaluated (Silberg, Lundberg & Musacchio, 1997; Charnock et al., 1999). According to literature DISCERN instrument can be applied by experienced users and providers of health information to discriminate between publications of high and low quality. Chance corrected agreement (weighted kappa) for the overall rating was found kappa = 0.53 (95% CI kappa = 0.48 to kappa = 0.59) among the expert panel. The instrument will also be of benefit to patients, though its use will be improved by training (Charnock et al., 1999).

### Quality analysis

Global Quality Score (GQS) is a system that evaluates the quality of online health information out of 5. 1 point indicates the lowest quality, 5 points the highest quality. According to this system, a source with a score of 1 is not of any quality for patients, while a source with a score of 5 is considered very high quality. In addition, 2 points: low quality, limited use; 3 points: medium quality, limited benefit; 4 points: good quality, useful (Gunduz et al., 2024). The Ensuring Quality Information for Patients (EQIP) is a tool that evaluates the quality and clarity of the relevant medical text. The 20 questions in this tool are answered as ‘yes’, ‘partially’ or ‘no’. According to the answers given, the quality of the information is determined with a score between 0 and 100. When answering the 20 questions in the scale, 1 point is given to the “yes” answer, 0.5 to the partially answer and 0 to the no answer. The obtained scores are added and divided by 20, and then those that do not apply are removed and multiplied by 100 ((X of Yes * 1) + (Y of Partly * 0.5) + (Z of No * 0))/(20 − (Q of does not apply))] *100 = % score) (Ladhar et al., 2022). According to the EQIP tool results, those between “0–25%” are evaluated as “severe problems with quality”, those between “26–50%” are evaluated as “serious problems with quality”, “51–75%” are evaluated as “good quality with minor problems”, and “76% to 100%” results are evaluated as “well written” (Moult, Franck & Brady, 2004). Reliability and validity assessments were made for the GQS and EQIP survey (Moult, Franck & Brady, 2004; Bernard et al., 2007). For example, The EQIP tool demonstrated strong validity, reliability, and utility in assessing the quality of a wide range of health information materials when employed by healthcare professionals and patient information managers. The internal consistency of the scale, as measured by Cronbach’s alpha, was 0.80. Inter-rater reliability was also satisfactory, with a mean agreement of 0.60 (Moult, Franck & Brady, 2004).

### Readability assessment

The responses given by AI chatbots to keywords were evaluated on two different websites that have the feature of calculating readability scores (http://readabilityformulas.com/, Calculator 1; https://www.online-utility.org/english/readability_test_and_improve.jsp, Calculator 2). The formulas used in text readability were Linsear Write (LW), Coleman-Liau Readability Index (CLI), Automated Readability Index (ARI), Simple Measure of Gobbledygook (SMOG), Gunning Fog Readability (GFOG), The Flesch Reading Ease Score (FRES) and Flesch Kincaid Grade Level (FKGL) (Gül et al., 2024; Özduran & Hanci, 2022; Hancı et al., 2024). Details on how readability was calculated with the formulas are given in Table 1. Final readability scores were recorded as median (minimum-maximum). The obtained responses were based on the sixth-grade It was analyzed with the readability level. Accordingly, the accepted average readability level is 80.0 for FRES and six for the other six formulas (Gül et al., 2024). The readability, quality and reliability level evaluation of the texts generated by artificial intelligence was carried out by two senior authors (EÖ and VH) with experience in the field of pain and the arithmetic average of the scores obtained in all three categories was taken.

**Table 1:** Readability tools, formulas and descriptions.

Readability index	Description	Formula
Gunning FOG (GFOG)	It was designed to assist American businesses in enhancing the readability of their written content and is applicable across various disciplines. It estimates the number of years of education required for a person to understand a given text.	G = 0.4 × (W/S + ((C * /W) × 100))
Flesch Reading Ease Score (FRES)	It was created to assess the readability of newspapers and is particularly effective for evaluating school textbooks and technical manuals. This standardized test is utilized by numerous US government agencies. The scores range from 0 to 100, with higher scores indicating greater ease of reading.	I = (206.835 − (84.6 × (B/W)) − (1.015 × (W/S)))
Flesch–Kincaid Grade Level (FKGL)	Part of the Kincaid Navy Personnel test collection, it was designed for technical documentation and is suitable for a wide range of disciplines. Delineates the academic capacity level imperative for grasping the written material	G = (11.8 × (B/W)) + (0.39 × (W/S)) − 15.59
Simple Measure of Gobbledygook (SMOG)	It is typically appropriate for middle-aged readers, ranging from 4th grade to college level. While it aims to test 100% comprehension, most formulas measure about 50–75% comprehension. It is most accurate when applied to documents that are at least 30 sentences long. It measures the number of years of education the average person needs to understand a text.	G = 1.0430 × √C + 3.1291
Coleman–Liau (CL) Score	It is designed for middle-aged readers, spanning from 4th grade to college level. The formula is based on text with a grade level range of 0.4 to 16.3 and is applicable to many industries. Evaluates the educational level required for understanding a text and offers an associated grade level in the US education system.	G = (−27.4004 × (E/100)) + 23.06395
Linsear Write (LW)	It was developed for the United States Air Force to assist in calculating the readability of their technical manuals. Offers an approximate assessment of the academic level needed to comprehend the text.	LW = (R + 3C)/S
		Result
		If > 20, divide by 2 If ≤ 20, subtract 2, and then divide by 2
Automated Readability Index (ARI)	The ARI (Automated Readability Index) has been utilized by the military for writing technical manuals. Its calculation provides the grade level required to comprehend the text. Assesses the scholastic rank in American educational institutions needed to be capable of comprehending written material. The greater the number of characters, the more complex the term.	ARI = 4.71 × l + 0.5 * ASL − 21.43

**Note:**

G, Grade level; B, number of syllables; W, number of words; S, number of sentences; I, Flesch index score; SMOG, simple measure of Gobbledygook; C, complex words (≥3 syllables); E, predicted cloze percentage = 141.8401 − (0.214590 × number of characters) + (1.079812 * S); C*, complex words with exceptions including, proper nouns, words made 3 syllables by addition of “ed” or “es”, compound words made of simpler words. ASL, the average number of sentences per 100 words R, the number of words ≤2 syllables.

### Statistical analysis

Data analysis was performed using SPSS Windows version 24.0 (SPSS Inc., Chicago, IL, USA). Frequency data are presented as numbers (n) and percentages (%), while continuous data are shown as medians (minimum-maximum). Fisher’s exact test and the Chi-square test were used to compare frequency variables, while the Mann-Whitney U and Wilcoxon tests were employed to compare continuous variables. To assess the consistency of the calculators, intraclass correlation coefficient (ICC) analysis was performed for each formula. Statistical significance was set at *p* < 0.05.

## Results

The first three keywords detected as a result of the Google Trend search were “Lower Back Pain”, “ICD 10 Low Back Pain”, and “Low Back Pain Symptoms”. The keyword “Pain in low back” was removed from the analysis because the keyword “Lower back pain” was present. The keywords “Low Back Pain ICD” and “Low Back Pain ICD 10 code” were removed from the analysis because the keyword “Low Back Pain ICD 10” was present. The keyword “Chronic Back Pain” was removed from the analysis because the keyword “Chronic Low Back Pain” was present. The keywords “Low back exercises” and “Back exercises” were removed from the analysis because the keyword “Low back pain exercises” was present. The keyword “Low Back Pain Kidney” was removed from the analysis because the keyword “Kidney Pain” was present. The keyword “Low Back Pain cause” was removed from the analysis because the keyword “Low Back Pain causes” was present. The keyword “what is Lower Back Pain” was removed from the analysis because the keyword “Lower back pain” was present. The keywords “Icd 10”, “lowback hip pain”, “hip pain” were removed because they were unrelated to the topic or showed different anatomical localizations. The study was organized on 13 keywords in the final. The full list of keywords is presented in Table 2. The United States, Puerto Rico, and Canada were determined to be the three countries with the highest searches for low back pain, respectively.

**Table 2:** Top 13 relevant keywords searched about low back pain across countries: 2004–2023 (based on Google Trends data).

Rank	Keyword	Category of the topic based on EQIP
1	Lower back pain	Condition or illness
2	Low back pain Icd	Condition or illness
3	Icd 10 low back pain	Condition or illness
4	Icd 10	Condition or illness
5	Low back pain symptoms	Condition or illness
6	Chronic low back pain	Condition or illness
7	Right low back pain	Condition or illness
8	Back pain exercises	Condition or illness
9	Chronic back pain	Condition or illness
10	Low back pain exercises	Condition or illness
11	Low back exercises	Condition or illness
12	Chronic low back pain	Condition or illness
13	Low back pain cause	Condition or illness
14	Low back pain causes	Condition or illness
15	Low left back pain	Condition or illness
16	Hip pain	Condition or illness
17	Low back hip pain	Condition or illness
18	Low back pain treatment	Discharge or aftercare
19	What is low back pain	Condition or illness
20	Low back muscle pain	Condition or illness
21	Sciatica	Condition or illness
22	Sciatica pain	Condition or illness
23	Kidney pain	Condition or illness
24	Low back pain kidney	Condition or illness
25	Exercises for low back pain	Condition or illness

**Note:**

EQIP, ensuring quality information for patients.

Keywords related to low back pain were entered into ChatGPT-4o, Perplexity and Google Gemini AI chatbots. The final readability scores of the responses given by these AIs were measured with two different programs, Calculator 1 and 2. The readability levels of the texts were evaluated by comparing them with the ability of a 6th grade reader to understand the text. The relevant results are given in Tables 3–6.

**Table 3:** Readability scores for Chatgpt-4o, Gemini, and Perplexity responses to the most frequently asked low back pain-related questions, and a statistical comparison of the text content to a 6th-grade reading level [Median (Minimum-Maximum)], using Calculator 1.

CALCULATOR 1 statistics	ChatGPT 4o^^^	Google Gemini^^^	Perplexity^^^	Chat GPT C6thGRL (P)* ^†^	Gemini C6thGRL (P)* ^†^	Perplexity C6thGRL (p)* ^†^	Between Chatgpt and Gemini^††^	Between Chatgpt and Perplexity^††^	Between Gemini and Perplexity^††^
FRES	45 (37–83)	56 (38–78)	30 (4–69)	**0.001**	**0.001**	**0.001**	0.174	**0.003**	**0.003**
GFOG	13.20 (8.10–17.90)	13.10 (10.50–18.20)	18.80 (11.50–56.00)	**0.001**	**0.001**	**0.001**	0.700	**<0.001**	**0.001**
FKGL	10.78 (4.91–13.05)	10.54 (6.47–16.16)	15.27 (8.64–51.88)	**0.002**	**0.001**	**0.001**	0.939	**<0.001**	**0.001**
CLI	13.72 (7.60–15.60)	11.16 (7.33–14.47)	16.17 (10.87–20.52)	**0.001**	**0.001**	**0.001**	**0.004**	**0.017**	**<0.001**
SMOG	9.68 (6–13.22)	9.44 (7.18–12.03)	13.62 (8.38–30.01)	**0.002**	**0.001**	**0.001**	0.590	**<0.001**	**<0.001**
ARI	12.11 (6.36–15.83)	12.03 (7.15–18.82)	17.24 (10.50–63.93)	**0.001**	**0.001**	**0.001**	0.999	**<0.001**	**0.001**
LW	10.02 (6.13–16.63)	12.37 (8.69–20.60)	16.15 (8.10–8*5*.50)	**0.001**	**0.001**	**0.001**	**0.008**	**<0.001**	**0.011**
Grade level	12.00 (7.00–14.00)	11.00 (8.00–16.00)	16.00 (10.00–45.00)	**0.001**	**0.001**	**0.001**	0.979	**<0.001**	**0.001**

**Notes:**

FRES, Flesch Reading Ease Score; FKGL, Flesch-Kincaid Grade Level; SMOG, Simple Measure of Gobbledygook; GFOG, Gunning FOG; CLI, Coleman-Liau Index; ARI, Automated Readability Index; LW, Linsear Write.

* C6thGRL(p), Comparison of the responses according to 6th grade reading level (p).

^ Median (minimum-maximum).

† Wilcoxon test.

†† Mann-Whitney U test.

*p* values in bold are statistically significant.

**Table 4:** Readability scores for ChatGPT-4o, Gemini, and Perplexity responses to the most frequently asked questions about low back pain, along with a statistical comparison of text content to a 6th-grade reading level [median (minimum-maximum)], using Calculator 2.

CALCULATOR 2 statistics	ChatGPT^^^	Gemini^^^	Perplexity^^^	ChatGPT C6thGRL (P)* ^†^	Gemini C6thGRL (P)* ^†^	Perplexity C6thGRL (p)* ^†^	Between ChatGPT and Gemini (p)^††^	Between ChatGPT and Perplexity (p)^††^	Between Perplexity and Gemini (p)††
FRES	43.48 (34.48–70.53)	49.18 (33.34–70.37)	29.14 (1.12–72.99)	**0.001**	**0.001**	**0.001**	**0**.158	**0.017**	**0.007**
GFOG	12.54 (7.12–14.17)	12.65 (8.72–17.54)	17.45 (9.81-55.70)	**0.001**	**0.001**	**0.001**	**0**.898	**<0.001**	**0.001**
FKGL	10.74 (6.20–12.24)	11.12 (6.94–16.25)	14.91 (6.43–52.38)	**0.001**	**0.001**	**0.001**	**0**.626	**<0.001**	**0.001**
CLI	13.65 (7.40–15.60)	11.21 (7.45–14.15)	16.21 (7.10–20.56)	**0.001**	**0.001**	**0.001**	**0.004**	**0.011**	**0.001**
SMOG	15.52 (8.20–13.76)	12.49 (9.03–14.71)	15.93 (10.14–32.75)	**0.001**	**0.001**	**0.001**	**0**.701	**<0.001**	**<0.001**
ARI	10.80 (4.70–12.87)	11.76 (5.65–18.33)	16.27 (4.75–63.64)	**0.002**	**0.001**	**0.001**	**0**.701	**<0.001**	**0.001**

**Notes:**

FRES, Flesch Reading Ease Score; GFOG, Gunning FOG; FKGL, Flesch-Kincaid Grade Level; CLI, Coleman-Liau Index; SMOG, Simple Measure of Gobbledygook; ARI, Automated Readability Index; LW, Linsear Write.

* C6thGRL(p), Comparison of the responses according to 6th grade reading level (p).

^ Median (minimum-maximum).

† Wilcoxon test.

†† Mann-Whitney U test.

*p* values in bold are statistically significant.

**Table 5:** Readability indices for ChatGPT-4o, Gemini, Perplexity responses on low back pain and statistical comparison of text content to 6th grade reading level (median [(minimum-maximum)]) using the average results obtained from Calculator 1 and Calculator 2.

Readability indexes	ChatGPT* ^^^	ChatGPT C6thGRL (p)** ^†^	Gemini* ^^^	Gemini C6thGRL (p)** ^†^	Perplexity* ^^^	Perplexity C6thGRL (p)** ^†^	Between ChatGPT and Gemini^††^	Between ChatGPT and Perplexity (p)^††^	Between Perplexity and Gemini (p)^††^
FRES	42.53 (35.74–76.77)	**0.001**	52.59 (35.67–72.01)	**0.001**	29.57 (2.56–71)	**0.001**	0.158	0.017	**0.007**
GFOG	12.54 (7.12–14.17)	**0.001**	12.88 (9.91–17.87)	**0.001**	18.13 (11.35–55.85)	**0.001**	0.898	**<0.001**	**0.001**
FKGL	10.84 (5.56–12.04)	**0.002**	10.68 (6.71–16.20)	**0.001**	15 (9.22–52.13)	**0.002**	0.626	**<0.001**	**0.001**
SMOG	11.15 (7.10–12.97)	**0.002**	10.97 (8.32–13.37)	**0.001**	14.69 (9.80–31.38)	**0.002**	0.701	**<0.001**	**<0.001**
CLI	13.65 (7.40–15.60)	**0.001**	11.22 (7.67–14.31)	**0.001**	16.19 (8.99–20.54)	**0.001**	**0.004**	0.011	**0.001**
ARI	11.49 (5.53–13.23)	**0.001**	11.91 (6.40–18.58)	**0.001**	16.72 (10.18–63.79)	**0.001**	0.701	**<0.001**	**0.001**

**Notes:**

Calculator 1: https://readabilityformulas.com/free-readability-formula-tests.php.

Calculator 2: https://www.online-utility.org/english/readability_test_and_improve.jsp.

FRES, Flesch Reading Ease Score; GFOG, Gunning FOG; FKGL, Flesch-Kincaid grade level; CLI, Coleman-Liau Index; SMOG, Simple Measure of Gobbledygook; ARI, Automated Readability Index; LW, Linsear Write.

* [(Calculator 1) + (Calculator 2)]/2.

** C6thGRL(p), Comparison of the responses according to 6th grade reading level (p).

^ Median (minimum-maximum).

† Wilcoxon test.

†† Mann-Whitney U test.

*p* values in bold are statistically significant.

**Table 6:** Comparison of JAMA, modified DISCERN, global quality scale (GQS) and EQIP ratings for the responses from ChatGPT-4o, Gemini, and Perplexity.

	ChatGPT *vs* Perplexity			ChatGPT *vs* Gemini			Perplexity *vs* Gemini		
	ChatGPT^^^	Perplexity^^^	*p*	ChatGPT^^^	Gemini^^^	*p*	Perplexity^^^	Gemini^^^	*p*
GQS, *n* (%)									
1-point	0 (0)	0 (0)	**0.005** *	0 (0)	0 (0)	**0.038** *	0 (0)	0 (0)	**<0.001** *
2-point	1 (7.7)	0 (0)		1 (7.7)	3 (23.1)		0 (0)	3 (23.1)	
3-point	7 (57.3)	0 (0		7 (57.3)	10 (76.9)		0 (0	10 (76.9)	
4-point	5 (38.5)	10 (76.9)		5 (38.5)	0 (0)		10 (76.9)	0 (0)	
5-point	0 (0)	3 (23.1)		0 (0)	0 (0)		3 (23.1)	0 (0)	
JAMA, *n* (%)									
0-point	13 (100)	0 (0)	**<0.001** *	13 (100)	12 (93.1)	**0.308** *	0 (0)	12 (93.1)	**<0.001** *
1-point	0 (0)	0 (0)		0 (0)	0 (0)		0 (0)	0 (0)	
2-point	0 (0)	0 (0)		0 (0)	0 (0)		0 (0)	0 (0)	
3-point	0 (0)	9 (69.2)		0 (0)	1 (7.7)		9 (69.2)	1 (7.7)	
4-point	0 (0)	4 (30.8)		0 (0)	0 (0)		4 (30.8)	0 (0)	
m DISCERN, *n* (%)									
1-point	3 (23.1)	0 (0)	**<0.001** *	3 (23.1)	10 (76.9)	**0.016** *	0 (0)	10 (76.9)	**<0.001** *
2-point	9 (69.2)	0 (0)		9 (69.2)	2 (15.4)		0 (0)	2 (15.4)	
3-point	1 (7.7)	9 (69.2)		1 (7.7)	1 (7.7)		9 (69.2)	1 (7.7)	
4-point	0 (0)	4 (30.8)		0 (0)	0 (0)		4 (30.8)	0 (0)	
5-point	0 (0)	0 (0)		0 (0)	0 (0)		0 (0)	0 (0)	
EQIP, *n* (%)									
Serious problems with qood quality	1 (7.7)	0 (0)	**<0.001** *	1 (7.7)	1 (7.7)	0.336	0 (0)	1 (7.7)	**<0.001** *
Good quality with minor problems	12 (92.3)	0 (0)		12 (92.3)	10 (76.9)		0 (0)	10 (76.9)	
Well written	0 (0)	13 (100)		1 (7.7)	2 (15.4)		13 (100)	2 (15.4)	

**Notes:**

EQIP, ensuring quality information for patients.

* Chi-Square test.

^ Median (minimum-maximum).

*p* values in bold are statistically significant.

### Assessment of readability across the three groups, utilizing average scores from calculators 1 and 2

In the analysis, the readability results of ChatGPT’s answers were found to be more difficult in Calculator 1 for the GFOG, FKGL, CLI and ARI readability formulas, and more difficult in Calculator 2 for the FRES, and SMOG readability formulas. Additionally, the readability results of Gemini’s and Perplexity’s answers were found to be more difficult in Calculator 1 for the GFOG and ARI readability formulas, and more difficult in Calculator 2 for the FRES, FKGL, CLI and SMOG readability formulas. When assessing the readability of responses among all three groups by averaging the outcomes from Calculator 1 and 2, significant differences emerged between specific groups. A significant difference (*p* = 0.004) was detected between ChatGPT-4o and Gemini in the CLI readability formula, but not in the other formulas. A significant difference was found between ChatGPT-4o and Perplexity in FOG, FKGL, SMOG and ARI readability formulas (*p* < 0.001). A significant difference was found between Gemini and Perplextiy in all readability formulas (*p* < 0.05) (Table 5). Based on the readability assessments, all readability metrics, excluding GFOG and ARI, are arranged in a hierarchy of readability from easiest to most difficult: Google Gemini, ChatGPT-4o, and Perplexity. Nonetheless, as per the GFOG and ARI readability metric, the order varies slightly: ChatGPT-4o, Google Gemini, Perplexity (Table 5).

### Assessing ChatGPT, Gemini, and perplexity responses based on the suggested reading level for sixth graders

When the median readability scores of all responses were compared to the sixth-grade reading level, a statistically significant difference was observed for all metrics (*p* < 0.001). Importantly, the readability of the responses exceeded the sixth-grade standard across all metrics. Similarly, statistically significant results were found when comparing the outcomes from Calculator 1 and Calculator 2, as well as the combined average of both calculators (*p* < 0.001) (Tables 3–5).

### Reliability and quality assessment

Perplexity’s answers achieved the top EQIP, JAMA, modified DISCERN and GQS scores (*p* < 0.001) (Table 6). According to these results, it can be said that Perplexity offers more reliable and quality data to its users.

### Intraclass correlation coefficients (ICC)

GFOG, FRES, CL, FKGL, ARI and SMOG scores were computed using two different calculators (https://www.online-utility.org/english/readability_test_and_improve.jsp, https://readabilityformulas.com/free-readability-formula-tests.php).

### ICC for ChatGPT

The intraclass correlation coefficient for FRES was 0.942, for FKGL was 0.876, for GFOG was 0.827, for CL was 0.951, for ARI was 0.827 and for SMOG was 0.852.

### ICC for Gemini

The intraclass correlation coefficient for FRES was 0.973, for KFGL was 0.985, for GFOG was 0.984, for CL was 0.972, for ARI was 0.978 and for SMOG was 0.976.

### ICC for Perplexity

The intraclass correlation coefficient for FRES was 0.930, for FKGL was 0.961, for GFOG was 0.955, for CL was 0.966, for ARI was 0.943 and for SMOG was 0.939.

### ICC for GQS, JAMA, mDISCERN and EQIP

The intraclass correlation coefficients were 0.915 for GQS, 0.981 for JAMA, 0.898 for mDISCERN 0.984 for EQIP.

According to these results, it can be said that there is a very strong correlation between the readability scores given by both calculators in our study and the quality and reliability survey answers given by both authors.

## Discussion

This study evaluated the quality, readability, and reliability of responses to frequently asked keywords about LBP provided by Perplexity, Gemini, and ChatGPT AI chatbots. Responses with reading levels higher than the 6th-grade reading level recommended by the U.S. Department of Health and Human Services and the National Institutes of Health were detected in all three AI chatbots. In addition, it was determined that Perplexity’s responses received higher results in reliability and quality analysis than other chatbots. To our knowledge, this study evaluated the information quality, reliability, and readability levels of responses to frequently asked keywords about LBP generated by three different popular AI chatbots and represents the first and pioneering research effort on this topic.

A key factor in understanding patient education materials is their readability. Complex and long sentences are known to undermine the reader’s confidence, making it difficult to learn health- related written texts. Furthermore, it has been shown that having eight to 10 words in a sentence facilitates the readability of health-related information (Gül et al., 2024). More readable texts will help create better health literacy, increasing patient compliance, reducing emergency care visits, and shortening hospital stays (Hancı et al., 2024).

In the literature, online information on LBP has been studied and shown to have readability levels that are more difficult than recommended. In a study on acute LBP, only three of 22 websites providing online information provided an acceptable readability score, while the readability levels of the other websites were reported to exceed the recommended level for the average person to understand (Hendrick et al., 2012). Another study investigated 72 websites on failed back spinal surgery and found that they were of low quality and content due to low JAMA and DISCERN scores (Guo et al., 2019). Studies frequently mentioned the detrimental effects of difficult readability and low quality and low reliability information on public health (Hendrick et al., 2012; Guo et al., 2019). AI chatbots have become a platform where a significant portion of patients seek answers to their medical questions due to their accessibility and ability to provide personalized answers. Some clinicians also consider these chatbots as a tool with the potential to improve patient education due to their broad knowledge base and ability to produce consistent and original answers (Ömür Arça et al., 2024). Therefore, our study aimed to evaluate the readability, quality and reliability parameters of the answers given by popular AI chatbots to keywords about LBP, not online websites.

There are also studies in the literature on the readability of the information provided by AI chatbots on LBP-related issues. Scaff et al. (2024), examined the answers to 30 questions about LBP to four different AI chatbots, namely ChatGPT 3.5, Bing, Bard and ChatGPT 4.0, and found that the answers were poor and could negatively impact patient understanding and behavior. The authors emphasized that poorly readable texts can challenge patients, potentially leading to misinformation, inappropriate care, and worsened health outcomes. Nian et al. (2024), found that ChatGPT’s answers to questions about lumbar spinal fusion and laminectomy were accurate but not specific enough and had readability appropriate for a slightly above average health literacy level. Each response resulted in a recommendation for further consultation with a healthcare provider, emphasizing to patients the value of physician consultation. There are studies in the literature showing that AI chatbots provide answers with high readability scores on different topics (Gül et al., 2024; Şahin et al., 2024).

In our study, it was determined similar to literature that the responses given by the AI chatbots to the most frequent keywords about LBP had readability levels higher than the 6th grade level recommended by the United States Department of Health and Human Services, the American Medical Association, and the National Institutes of Health. The easiest readability was determined in Google Gemini, while the most difficult readability was determined in Perplexity. Developing AI chatbots with appropriate readability will help patients trying to access health-related online information to reach more understandable information.

In our study, not only the readability of AI chatbots but also their quality and reliability were tested. In the study examining the responses given by four different chatbots, ChatGPT, BARD, Gemini and Copilot, to questions about palliative care in the literature, it was reported that none of the ChatGPT responses met the JAMA criteria. In addition, it was determined that mDISCERN and JAMA scores were the highest in Perplexity, and GQS scores were the highest in Gemini (Hancı et al., 2024). In the study conducted by Casciato et al. (2024) on the information given by AI chatbots on foot and ankle surgery, they determined that they had low reliability and accuracy due to low JAMA and DISCERN scores. There are some studies in the literature showing that Perplexity produces answers with high DISCERN and JAMA scores (Gül et al., 2024; Hancı et al., 2024). In our study, high mDISCERN, JAMA, GQS and EQIP scores were detected in Perplexity. These scores were significantly lower in other AI chatbots. In the future, new chatbots or new versions of existing chatbots can be produced, and these adjustments can be made to provide higher quality and more reliable information to those requesting online health-related information. Additionaly, it is an undeniable fact that none of the information provided by these chatbots can replace a face-to-face medical consultation.

### Potential implications for clinical practice and future studies

This study highlighted the potential and limitations of AI chatbots’ responses regarding LBP. Although it is emphasized that the answers obtained help to provide significant gains to public health by providing reliable and quality content, the fact that answers with high readability scores for patients with average health literacy may cause patients to make incorrect or incompletely informed health decisions and receive inappropriate or delayed health care. Additionally, this study can provide inspiration for future studies on improving the algorithms and responses of AI chatbots and provide guidance for possible policies regarding AI’s appropriate information delivery on patient education and health literacy.

### Limitations of the study

We can list the limitations in our study as follows. The study we planned using the 25 most popular keywords offered by Google regarding low back pain can be made more comprehensive with studies to be produced using more keywords in the future. Another limitation is the presence of only English-language keywords in our study. In addition, studies to be conducted with other chatbots other than Gemini, ChatGPT and Perplexity will reveal the functioning of different artificial intelligence models. In our study, we evaluated the responses given by chatbots to the keywords detected on a day in May 2024. This situation shows that different keywords that can be obtained on another date may yield different study results.

### Strength of the study

Our study is the first to demonstrate not only the readability but also the quality and reliability of AI chatbots on LBP. Unlike many other study methodologies, the fact that we evaluated the responses of multiple popular AI chatbots, not just a single AI chatbot, can be considered another strength of the study.

## Conclusion

AI chatbots such as ChatGPT, Perplexity and Gemini are increasingly performing well in providing medical information. This may provide an opportunity to raise awareness and improve patient satisfaction on issues related to LBP. Despite this, there are still some concerns about the readability, quality and reliability assessment results of AI chatbots. In our study, it has been determined that the answers given by AI chatbots to keywords about LBP are difficult to read and have low reliability and quality assessment. In the future, the information provided in AI chatbots will be presented through an expert team review and texts with appropriate and understandable readability will positively affect public health.

## Supplemental Information

10.7717/peerj.18847/supp-1 Supplemental Information 1 Raw data.

10.7717/peerj.18847/supp-2 Supplemental Information 2 Codebook.

## Additional Information and Declarations

### Data Availability

The following information was supplied regarding data availability: The raw data is available in the Supplemental File.
