# Supplementary material for: Assessing the readability, quality and reliability of responses produced by ChatGPT, Gemini, and Perplexity regarding most frequently asked keywords about low back pain
Source: PeerJ. 2025 Jan 22;13:e18847. doi: 10.7717/peerj.18847 (PMC11760201; doi:10.7717/peerj.18847)
Supplement: Supplemental Information 2 [file peerj-13-18847-s002.docx]

Codebook

- fleshgeminic1: Score obtained using the FRES readability formula with the help of calculator 1 of Gemini's answer
- fleshgeminic2: Score obtained using the FRES readability formula with the help of calculator 2 of Gemini's answer
- fleshgeminitpl: Average result of both calculators in Gemini FRES results
- gfoggeminic1: Score obtained using the GFOG readability formula with the help of calculator 1 of Gemini's answer
- gfoggeminic2: Score obtained using the GFOG readability formula with the help of calculator 2 of Gemini's answer
- gfoggeminitpl: Average result of both calculators in Gemini GFOG results
- fkglgeminic1: Score obtained using the FKGL readability formula with the help of calculator 1 of Gemini's answer
- fkglgeminic2: Score obtained using the FKGL readability formula with the help of calculator 2 of Gemini's answer
- fkglgeminitpl: Average result of both calculators in Gemini FKGL results
- clgeminic1: Score obtained using the CL readability formula with the help of calculator 1 of Gemini's answer
- clgeminic2: Score obtained using the CL readability formula with the help of calculator 2 of Gemini's answer
- cltgeminitpl: Average result of both calculators in Gemini CL results
- smoggeminic1: Score obtained using the FRES readability formula with the help of calculator 1 of Gemini's answer
- smoggeminic2: Score obtained using the FRES readability formula with the help of calculator 2 of Gemini's answer
- smoggeminitpl: Average result of both calculators in Gemini SMOG results
- arigeminic1: Score obtained using the ARI readability formula with the help of calculator 1 of Gemini's answer
- arigeminic2: Score obtained using the ARI readability formula with the help of calculator 2 of Gemini's answer
- arigeminitpl: Average result of both calculators in Gemini ARI results
- lwgeminic1: Score obtained using the LW readability formula with the help of calculator 1 of Gemini's answer
- gradelevelgeminic1: Grade Level Score obtained using calculator 1 of Gemini's answer
- readlevelgeminic1: Reading Level Score obtained using calculator 1 of Gemini's answer
- readagegeminic1: Reading Age Score obtained using calculator 1 of Gemini's answer
- GQSgeminiA: Gemini’s Global Quality Score Final Score for the Evaluator Autor A
- GQSgeminiB: Gemini’s Global Quality Score Final Score for the Evaluator Autor B
- GQSgemini: Gemini’s Average Global Quality Score Final Score for the Evaluator Autor A and B
- Gqsgemd: Global Quality Score Assesment
- JAMAGeminiA: Gemini’s JAMA Final Score for the Evaluator Autor A
- JAMAGeminiB: Gemini’s JAMA Final Score for the Evaluator Autor B
- JAMAgemini: Gemini’s Average JAMA Final Score for the Evaluator Autor A and B
- jamagemd3: JAMA Assesment
- DISCERNgeminiA: Gemini’s DISCERN Final Score for the Evaluator Autor A
- DISCERNgeminiB: Gemini’s DISCERN Final Score for the Evaluator Autor B
- DISCERNgemini: : Gemini’s Average DISCERN Final Score for the Evaluator Autor A and B
- Discerngemd: DISCERN Assesment
- EQIPGeminiA: Gemini’s EQIP Final Score for the Evaluator Autor A
- EQIPGeminiB: Gemini’s EQIP Final Score for the Evaluator Autor B
- EQIPGemini: Gemini’s Average EQIP Final Score for the Evaluator Autor A and B
- EQIPGemini5: EQIP Assesment
- Fleshreadingeasecg: Score obtained using the FRES readability formula with the help of calculator 1 of ChatGPT's answer
- flechreadingease2cg: Score obtained using the FRES readability formula with the help of calculator 2 of ChatGPT's answer
- FLESHGPTTPL: Average result of both calculators in ChatGPT FRES results
- Gunningfogcg: Score obtained using the GFOG readability formula with the help of calculator 1 of ChatGPT's answer
- gunningfog2cg: Score obtained using the GFOG readability formula with the help of calculator 2 of ChatGPT's answer
- GFPGGPTTPL: Average result of both calculators in ChatGPT GFOG results
- Fleschkincaidgradecg: Score obtained using the FKGL readability formula with the help of calculator 1 of ChatGPT's answer
- fleschkncaidgrade2cg: Score obtained using the FKGL readability formula with the help of calculator 2 of ChatGPT's answer
- FKINGGPTTPL: Average result of both calculators in ChatGPT FKGL results
- Colemanliaucg: Score obtained using the CL readability formula with the help of calculator 1 of ChatGPT's answer
- colemanliau2cg: Score obtained using the CL readability formula with the help of calculator 2 of ChatGPT's answer
- CLGPTTPL: Average result of both calculators in ChatGPT CL results
- Smogcg: : Score obtained using the FRES readability formula with the help of calculator 1 of ChatGPT's answer
- smog2cg: Score obtained using the FRES readability formula with the help of calculator 2 of ChatGPT's answer
- SMOGPPTTPT: Average result of both calculators in ChatGPT SMOG results
- Automatedreadabilitycg: Score obtained using the ARI readability formula with the help of calculator 1 of ChatGPT's answer
- automatedreadability2cg: Score obtained using the ARI readability formula with the help of calculator 2 of ChatGPT's answer
- ARIGPTTPL: Average result of both calculators in ChatGPT ARI results
- Linsearwritecg: Score obtained using the LW readability formula with the help of calculator 1 of ChatGPT's answer
- Grdelevcg: Grade Level Score obtained using calculator 1 of ChatGPT's answer
- Readlevcg: Reading Level Score obtained using calculator 1 of ChatGPT's answer
- Readsagecg: Reading Age Score obtained using calculator 1 of ChatGPT's answer
- GQScgA: ChatGPT’s Global Quality Score Final Score for the Evaluator Autor A
- GQScgB: ChatGPT’s Global Quality Score Final Score for the Evaluator Autor B
- GQScg: ChatGPT’s Average Global Quality Score Final Score for the Evaluator Autor A and B
- GQScg3: Global Quality Score Assesment
- JAMAcgA: ChatGPT’s JAMA Final Score for the Evaluator Author A
- JAMAcgB: ChatGPT’s JAMA Final Score for the Evaluator Author B
- JAMAcg: ChatGPT’s Average JAMA Final Score for the Evaluator Autor A and B
- jamacg3: JAMA Assesment
- DISCERNcgA: ChatGPT’s DISCERN Final Score for the Evaluator Autor A
- DISCERNcgB: ChatGPT’s DISCERN Final Score for the Evaluator Autor B
- DISCERNcg: ChatGPT’s DISCERN Final Score for the Evaluator Autor A and B
- DISCERNcg5: DISCERN Assesment
- EQIPcgA: ChatGPT’s EQIP Final Score for the Evaluator Autor A
- EQIPcgB: ChatGPT’s EQIP Final Score for the Evaluator Autor B
- EQIPcg: ChatGPT’s Average EQIP Final Score for the Evaluator Autor A and B
- EQIPcg5: EQIP Assesment
- Fleschreadingeaseperplexity: Score obtained using the FRES readability formula with the help of calculator 1 of Perplexity's answer
- fleschreadingease2perplexity: Score obtained using the FRES readability formula with the help of calculator 2 of Perplexity's answer
- PRPLXFRETPL: Average result of both calculators in Perplexity FRES results
- Gunningfogperplexity: Score obtained using the GFOG readability formula with the help of calculator 1 of Perplexity's answer
- Gunningfoxindexperplexity: Score obtained using the GFOG readability formula with the help of calculator 2 of Perplexity's answer
- PRPLGFOGTPL: Average result of both calculators in Perplexity GFOG results
- Fleschkincaidgradeperplexity: Score obtained using the FKGL readability formula with the help of calculator 1 of Perplexity's answer
- Fleschkincaidperplexity: Score obtained using the FKGL readability formula with the help of calculator 2 of Perplexity's answer
- PRPLXFKINTPL: Average result of both calculators in Perplexity FKGL results
- Colemanliauperplexity: Score obtained using the CL readability formula with the help of calculator 1 of Perplexity's answer
- Colemanliauindexperplexity: Score obtained using the CL readability formula with the help of calculator 2 of Perplexity's answer
- PREPLCLTPT: Average result of both calculators in Perplexity CL Results
- SMOGindexperplexity: Score obtained using the SMOG readability formula with the help of calculator 1 of Perplexity's answer
- SMOGperplexity: Score obtained using the SMOG readability formula with the help of calculator 2 of Perplexity's answer
- PRPLXSMOGTPL: Average result of both calculators in Perplexity SMOG Results
- ARIperplexity: Score obtained using the ARI readability formula with the help of calculator 1 of Perplexity's answer
- ARI2perplexity: Score obtained using the ARI readability formula with the help of calculator 2 of Perplexity's answer
- PRPLXARITPL: Average result of both calculators in Perplexity ARI Results
- Linsearwriteperplexity: Score obtained using the LW readability formula with the help of calculator 1 of Perplexity's answer
- Gradelevelperplexity: Grade Level Score obtained using calculator 1 of Perplexity's answer
- Readinglevelperplexity: Reading LEvel Score obtained using calculator 1 of Perplexity's answer
- Readersageperplexity: Readers Age Score obtained using calculator 1 of Perplexity's answer
- GQSperplexityA: Perplexity’s Global Quality Score Final Score for the Evaluator Autor A
- GQSperplexityB: Perplexity’s Global Quality Score Final Score for the Evaluator Autor B
- GQSperplexity: Perplexity’s Average Global Quality Score Final Score for the Evaluator Autor A and B
- GQSperplexity3: Global Quality Score Assesment
- JAMAperplexityA: Perplexity’s JAMA Final Score for the Evaluator Author A
- JAMAperplexityB: Perplexity’s JAMA Final Score for the Evaluator Author B
- JAMAperplexity: Perplexity’s Average JAMA Final Score for the Evaluator Autor A and B
- JAMAperplexity3: JAMA Assesment
- DiscernperplexityA: Perplexity’s DISCERN Final Score for the Evaluator Autor A
- DiscernperplexityB: Perplexity’s DISCERN Final Score for the Evaluator Autor B
- Discernperplexity: Perplexity’s DISCERN Final Score for the Evaluator Autor A and B
- Discernperplexity5: DISCERN Assesment
- EQIPperplexityA: Perplexity’s EQIP Final Score for the Evaluator Autor A
- EQIPperplexityB: Perplexity’s EQIP Final Score for the Evaluator Autor B
- EQIPperplexity: Perplexity’s Average EQIP Final Score for the Evaluator Autor A and B
- EQIPperplexity5: EQIP Assesment
- FLESH6: 6th class level of FRES score
- GFOG6: 6th class level of GFOG score
- FKING6: 6th class level of FKGL score
- CL6: 6th class level of CL score
- SMOG6: 6th class level of SMOG score
- ARI6: 6th class level of ARI score
- LINSER6: 6th class level of LW score
- GRDLVL6: 6th class level of Grade LEvel
- READLVL6: 6th class level of Read Level
- READAGE6: 6th class level of readers Age
- Readlevelgeminic1

0: easy to read
1: fairly easy to read.

2: standard / average

3: fairly difficult to read

4: difficult to read

5: very difficult to read

6: impossible to comprehend

7: Extremely Difficult

8: Professional

9: Somewhat Difficult

- Readagegeminic1

1: 8-9 yrs. old (Fourth and Fifth graders)
2: 10-11 yrs. olds (Fifth and Sixth graders)
3: 11-13 yrs. old (Sixth and Seventh graders
4: 12-14 yrs. old (Seventh and Eighth graders)

5: 13-15 yrs. old (Eighth and Ninth graders)

6: 14-15 yrs. old (Ninth to Tenth graders

7: 15-17 yrs. old (Tenth to Eleventh graders)

8: 17-18 yrs. old (Twelfth graders)

9: 18-19 yrs. old (college level entry)

10: 21-22 yrs. old (college level)

11: 23+ year olds

12: College graduate

- Gqsgemd

1: low quality
2: medium quality
3: high quality

- jamagemd3
  1:Insufficient data

2: Partially sufficient data

3: Completely sufficient data

- Discerngemd
  1: very poor
  2: poor

3: fair

4: good

5: excellent

- EQIPGemini5

1: severe problems with quality
2: serious problems with quality
3: good quality with minor problems
4: well written

- Readlevcg

0: easy to read
1: fairly easy to read.

2: standard / average

3: fairly difficult to read

4: difficult to read

5: very difficult to read

6: impossible to comprehend

7: Extremely Difficult

8: Professional

- Readsagecg

1: 8-9 yrs. old (Fourth and Fifth graders)
2: 10-11 yrs. olds (Fifth and Sixth graders)
3: 11-13 yrs. old (Sixth and Seventh graders
4: 12-14 yrs. old (Seventh and Eighth graders)

5: 13-15 yrs. old (Eighth and Ninth graders)

6: 14-15 yrs. old (Ninth to Tenth graders

7: 15-17 yrs. old (Tenth to Eleventh graders)

8: 17-18 yrs. old (Twelfth graders)

9: 18-19 yrs. old (college level entry)

10: 21-22 yrs. old (college level)

11: 23+ year olds

12: College graduate

- GQScg3

1: low quality
2: medium quality
3: high quality

- jamacg3

1:Insufficient data

2: Partially sufficient data

3: Completely sufficient data

- DISCERNcg5

1: very poor
2: poor

3: fair

4: good

5: excellent

- EQIPcg5

1: severe problems with quality
2: serious problems with quality
3: good quality with minor problems
4: well written

- readinglevelperplexity

0: easy to read
1: fairly easy to read.

2: standard / average

3: fairly difficult to read

4: difficult to read

5: very difficult to read

6: impossible to comprehend

7: Extremely Difficult

8: Professional

- readersageperplexity

1: 8-9 yrs. old (Fourth and Fifth graders)
2: 10-11 yrs. olds (Fifth and Sixth graders)
3: 11-13 yrs. old (Sixth and Seventh graders
4: 12-14 yrs. old (Seventh and Eighth graders)

5: 13-15 yrs. old (Eighth and Ninth graders)

6: 14-15 yrs. old (Ninth to Tenth graders

7: 15-17 yrs. old (Tenth to Eleventh graders)

8: 17-18 yrs. old (Twelfth graders)

9: 18-19 yrs. old (college level entry)

10: 21-22 yrs. old (college level)

11: 23+ year olds

12: College graduate

- GQSperplexity3

1: low quality
2: medium quality
3: high quality

- JAMAperplexity3

1:Insufficient data

2: Partially sufficient data

3: Completely sufficient data

- Discernperplexity5

1: very poor
2: poor

3: fair

4: good

5: excellent

- EQIPperplexity5

1: severe problems with quality
2: serious problems with quality
3: good quality with minor problems
4: well written

- READLVL6

0: easy to read
1: fairly easy to read.

2: standard / average

3: fairly difficult to read

4: difficult to read

5: very difficult to read

6: impossible to comprehend

7: Professional

- READAGE6

1: 8-9 yrs. old (Fourth and Fifth graders)
2: 10-11 yrs. olds (Fifth and Sixth graders)
3: 11-13 yrs. old (Sixth and Seventh graders
4: 12-14 yrs. old (Seventh and Eighth graders)

5: 13-15 yrs. old (Eighth and Ninth graders)

6: 14-15 yrs. old (Ninth to Tenth graders

7: 15-17 yrs. old (Tenth to Eleventh graders)

8: 17-18 yrs. old (Twelfth graders)

9: 18-19 yrs. old (college level entry)

10: 21-22 yrs. old (college level)

11: 23+ year olds

12: College graduate
